# Comparative performance of hybrid model based on discrete wavelet transform and ARIMA models in prediction incidence of COVID-19

**DOI:** 10.1016/j.heliyon.2024.e33848

**Published:** 2024-06-27

**Authors:** Kourosh Holakouie-Naieni, Mojtaba Sepandi, Babak Eshrati, Shahrzad Nematollahi, Yousef Alimohamadi

**Affiliations:** a Department of Epidemiology and Biostatistics, School of Public Health, Tehran University of Medical Sciences, Tehran, Iran; b Health Research Center, Life Style Institute, Baqiyatallah University of Medical Sciences, Tehran, Iran; c Department of Community Medicine, School of Medicine, Preventive Medicine and Public Health Research Center, Psychosocial Health Research Institute, Iran University of Medical Sciences, Tehran, Iran

**Keywords:** Hybrid model, DWT, ARIMA, Prediction, COVID-19

## Abstract

**Objective:**

Public health surveillance is an important aspect of outbreak early warning based on prediction models. The present study compares a hybrid model based on discrete wavelet transform (DWT) and ARIMA (Autoregressive Integrated Moving Average) for predicting incidence cases due to COVID-19.

**Methods:**

In the current cross-sectional stuady based on time-series data, the incidence data for confirmed daily cases of COVID-19 from February 26, 2019, to April 25, 2022, were used. A hybrid model based on DWT and ARIMA and a pure ARIMA model were used to predict the trend. All analyzes were performed by MATLAB 2018, stata 2015, and Excel 2013 computer software.

**Results:**

Compared to the ARIMA model, the prediction results of the hybrid model were closer to the actual number of incident cases. The correlation between predicted values by the hybrid model with real data was higher than the correlation between predicted values by the ARIMA model with actual data.

**Conclusions:**

Discreet Wavelet decomposition of the dataset was combined with an ARIMA model and showed better performance in predicting the future trend.

## 1 Introduction

An important issue in the field of public health surveillance is early warning, based on the idea of predicting the present data value from past data, and then comparing the prediction with the observed value. A number of algorithms with different performance have been applied, including ARIMA (Autoregressive Integrated Moving Average), SARIMA (Seasonal Autoregressive Integrated Moving Average) and CUSUM (Cumulative sum) [[1], [2], [3]]. Such algorithms present some limitations, including non-stationarity in data, normal distribution, different scales of periodic changes, including daily, weekly, monthly, and/or annual periods, changes due to non-disease-related events (e.g., holidays) [4]. Time series analysis is the examination of the pattern of data in the past with the aim of predicting future values [5]. One of the most common models used in the time-series data analysis is the Box-Jenkins based ARIMA model, which covers different patterns of time series data [6,7]. However, when data is not following the time linearly, Box-Jenkins methodology would be inappropriate [8] while the Real-world datasets are often noisy and lacks the normal distribution. Discrete wavelet transform (DWT) is one of the mathematical methods that can be used to identify and predict occurrence of events without the mentioned assumptions. DWT is a type of the signal processing methods that extracts important features by decomposing the time-series data and removing the redundant data points and noise. One of the important capabilties of this method is the ability to deal with large non-stationary time-series data. This method can be used to identify and predict all types of outbreaks and even bioterrorism attacks [9].

The present study aimed to compare a hybrid model based on DWT and ARIMA for making the prediction of incidence cases due to COVID-19. We used a dataset of daily new cases of COVID-19 occurred in Tehran city. We hypothesized that the performance of the hybrid model would be superior to the pure ARIMA model.

## 2 Materials and methods

### 2.1 Data

The data included confirmed cases of COVID-19 on a daily basis from February 26, 2019, to April 25, 2022 in Tehran city that were recorded by the Iran University of Medical Sciences (IUMS). The mentioned data were obtained from three sources that included medical care monitoring center (MCMC), the health vice chancellor included positive PCR cases, and the data of the treatment vice chancellor included confirmed cases by PCR and patients based on the clinical definition of COVID-19.

### 2.2 Data analysis

A hybrid model based on DWT and ARIMA and a pure ARIMA model were used to predict the trend of the disease.

### 2.3 Discrete wavelet transform

**Fig. 1:**
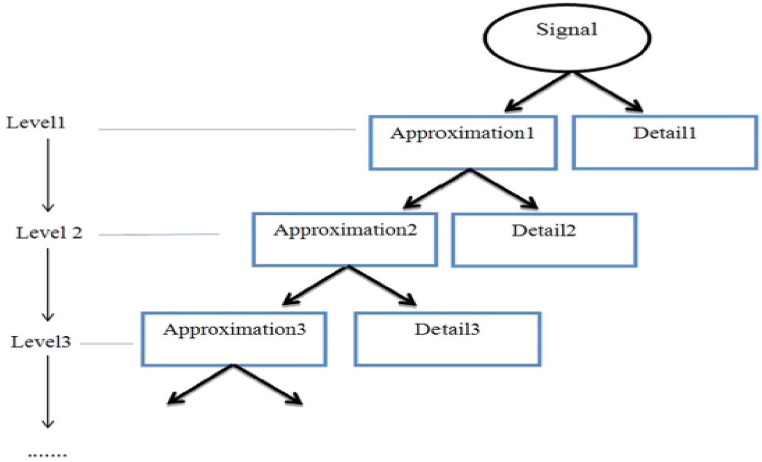
The process of discrete wavelet transform [9].

The first step in the wavelet transformation is selecting the parent wavelet function. There are different types of these functions, the most important of which are: Haar, Dabishes, Mexican Hat, Symlet, Morelet, and Mayer wavelets [10]. The two main parts of DWT are approximation and details coefficients. Approximation refers to the low-frequency components and the large scale of the signal, and detail refers to the high-frequency components and the low scale of the signal [11]. In this method, firstly, the time-series data converted to frequency. Then, it passes through the wavelet transform filters, resulting in this step of producing detail and approximation coefficients of Step 1. In the next step, this process was repeated on the approximation coefficient obtained from the first step, and the approximation coefficients and details obtained from Step 2 will be produced again. This process was continued until the approximation coefficients finally approached to zero. Analysis was performed in dyadic form [12]. The schematic structure of the DWT is shown in Fig. 1.

### 2.4 ARIMA model

In this model, the data were first checked in terms of stationarity in mean and variance. Then, the appropriate pattern for the data, based on the type of data will be identified. To select the best forecasting model, time-series graphs and statistics such as the partial autocorrelation function (PACF) the autocorrelation function (ACF), and the Akaike information criterion (AIC) statistic were used. The best model for the prediction is the model with minimum AIC. For assessing the fitness of the selected models for prediction, the tests of randomness on residuals such as Ljung - Box statistic, McLeod - Li statistic, Turning points, Diff sign points, Rank test statistic, Jarque-Bera test statistic (for normality) and the schematic checking of the residual graph were used [13].

### 2.5 Hybrid model for prediction

Based on this model, the studied time-series data will be decomposed into different levels by the desaired wavelet function. In the current study due to simplicity the Haar wavelet was used. In the next step, the ARIMA model will predict all detail coefficient levels and the last approximation coefficient. For example, if the signal decomposition goes up to three levels, the final prediction model will be calculated from the approximations (Aj) and detail (Dj) coefficients that are summed to obtain forecasted data (f (t)), using the following formula:

$$\hat{f\left(t\right)}=\hat{A3}+\hat{D1}+\hat{D2}+\hat{D3}$$

where, A3 is the predicted value of the approximation coefficient at the third level, and D is the predicted value of details coefficient values for each level [14].

### 2.6 Performance evaluation methods

To evaluate the performance of the methods, the first 75 % of the data was considered as the ‘Test’ and the remaining as the ‘Trained’ [15]. In other words, 300 days of data were used in this analysis. The data of 250 days was used to predict the next 50 days, and then the predicted value was graphed against with the actual value. The cross-correlation between the predicted values and the actual data was calculated. High correlation values between the observed and expected values in each lag indicated the better performance of the model. All analyzes were performed by MATLAB 2018, stata 2015, and Excel 2013 software.

## 3 Results

### 3.1 Descriptive results

The minimum and maximum number of daily cases from outpatient centers was 1 and 367, respectively. The maximum number of daily deaths was 47. The highest number of hospitalized cases was 606, and the highest case fatality rate (CFR) was 24.47 %. (Table 1).

**Table 1:** Descriptive indicators of reported cases of Covid-19 in the studied period.

Variable	Minimum	Maximum	Median (IQR)
Reported case^a^	1	367	45 (70–27)
Reported deaths	0	47	45 (8–2)
Hospitalization	32	606	169 (225–123)
Case Fatality Rate(%)	0	24.47	2.7 (4.40–1.5)

a Outpatient cases.

**Fig. 2:**
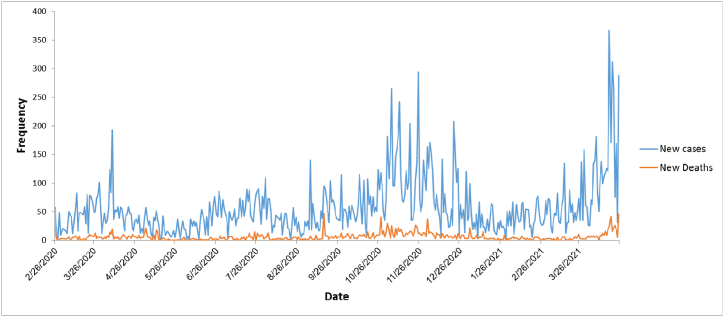
The trend of reported new cases and deaths of COVID-19 from Iran University of medical sciences.

**Fig. 3:**
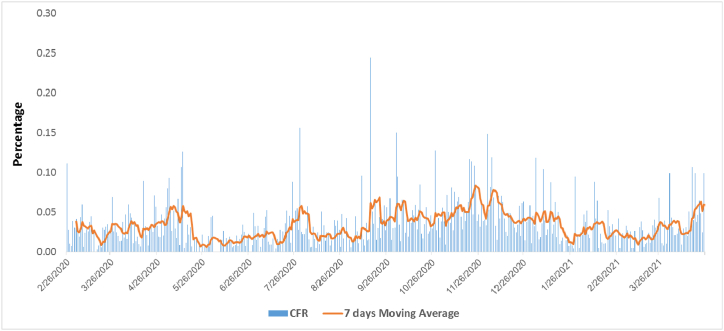
The trend of case fatality rate (CFR) of COVID-19 from Iran University of medical sciences.

Fig. 2 show the trend of reported new cases and deaths during the understudied period. Fig. 3 shows the trend of CFR due to COVID-19.

### 3.2 The performance of different methods in predicting cases

#### 3.2.1 ARIMA method

**Fig. 4:**
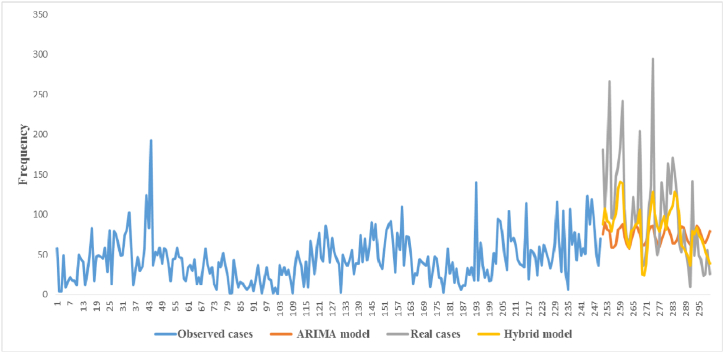
Predicted new cases based on hybrid and ARIMA models vs actual cases of COVID-19.

Three hundred days of data were used in this analysis. The data of 250 days was used to predict the next 50 days, and then the predicted value was graphed against with the actual value. ARIMA model (7,1,6) was used to predict new cases. In Fig. 4, the number of predicted cases for 50 days (brown line) and the actual number of observed cases for 50 days (gray line) are shown. This graph shows the difference between the real value and the predicted value by the ARIMA model.

#### 3.2.2 Hybrid model (combination of ARIMA and DWT)

**Fig. 5:**
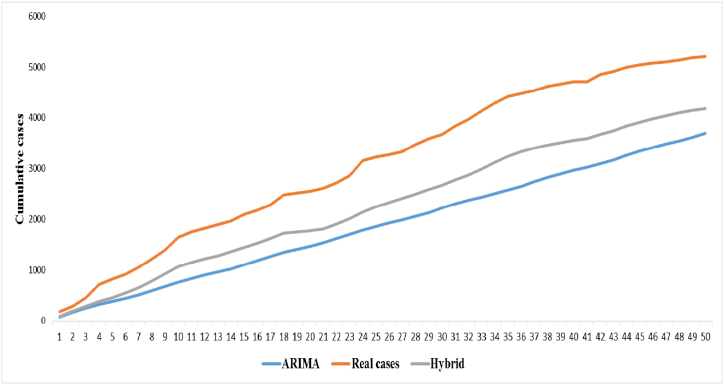
Comparison of Prediction of new cases (cumulatively) using Hybrid and ARIMA-based models and actual cases of COVID-19.

The Prediction results based on the hybrid model compared to the ARIMA model are displayed in Fig. 4(orange line). This graph shows the difference between the real value and the predicted value by the hybrid model**.** Compared to the ARIMA model, it is clear that the hybrid model results are closer to the actual number of cases (Fig. 5).

#### 3.2.3 Cross-correlation of prediction with real data

**Fig. 6:**
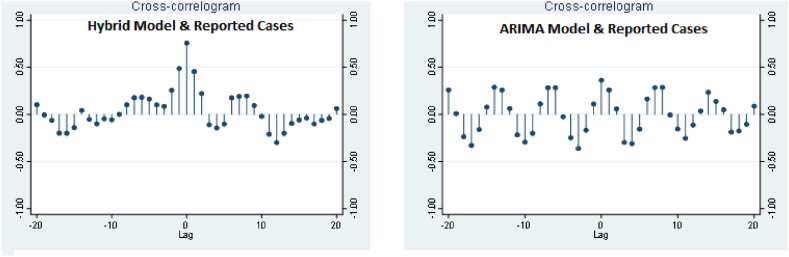
Cross-correlation of predicted with reported values of COVID-19 based on hybrid and ARIMA models.

Cross-correlation of predicted with reported values based on hybrid and ARIMA models was showed ih Fig. 6. According to this figure, the correlation coefficient between predicted values by the hybrid model with real data was higher than the correlation coefficient between predicted values by the ARIMA model and actual. In other words, the correlation coefficients (r) between the predicted values and the actual values in the hybrid model were higher than the ARIMA model, which indicates the better performance of this model.

## 4 Discussion

The present study was the first study in Iran aimed to use a hybrid model based on the DWT along with an ARIMA model on prediction incidence of COVID-19. Accurate prediction of the disease trend is a crucial step to detect outbreaks. The results of the present study, in line with the study of Singh et al., in 2020 [14] showed that the discrete wavelet transform method, or paying attention to lack of the need to set up a default for the normal distribution of data and data stationarity, compared to ARIMA method has more performance in predicting the future trends of morbidity and mortality from COVID-19. Several studies have been conducted on predicting the cases of COVID-19 in Iran using different models [[16], [17], [18]]. But a similar study was not found in Iran. Omidi et al. showed the appropriate performance of an Artificial neural network in the prediction of COVID-19 cases [18]. Aghakhani et al. showed that the XGBoost, LightGBM, and random forest have a relatively high predictive performance in prediction of mortality of COVID-19 [19] and sabetian et al. report good advantages of machine-learning methods for detection of COVID-19 Requiring Intensive Care [17].

Predicting the trend of diseases is an important issue in public health, which contributes in timely control of outbreaks [20]. Public health practitionners can prevent outbreaks via timely decisions and consequent rapid responses [21]. One of the strengths of the present study is the simultaneous use of two prediction models and the use of real data instead of simulated data. Few studies have simultaneously used such a methodology to evaluate methods for predicting the course of the diseases [8,22]. Their results show the better performance of the hybrid model based on the wavelet compared to the ARIMA model. Prediction of cases using this model may help policymakers to use of preventive measures before any catastrophic situation. It is worth noting that all forecasts based on models are not definitive. Because the future trend of outbreaks is affected by various factors, including the level of herd immunity, the percentage of susceptible population, vaccination coverage, and other mass preventive measures such as social distancing rules [23]. Crosscorrelation analysis reveal that the predicted values based on the hybrid model were more correlated with real reported values compared to the ARIMA model. One of the limitations of the current study, especially in the case of the discrete wavelet transform method, was the need for long-term data, so in the short-term data, the results must be interpreted with caution.

## 5 Conclusion

In this paper, the performance of the hybrid (DWT-ARIMA) and ARIMA models was investigated using COVID-19 data. Discreet Wavelet decomposition of the dataset was combined with an ARIMA model in order to develop a better hybrid model to forecast future cases accurately. The forecast obtained by the hybrid Wavelet-ARIMA model showed better performance compared to the ARIMA model. So the prediction by this technique could benefit health authorities to take control measures.

## Source of funding

This research supported by Iranian 10.13039/501100012155National Institute for Medical Research Development.

## Ethical approval

This manuscript approved by the Research Ethics Committees of National Institute for Medical Research Development by approval id **IR.NIMAD.REC.1399.305.**

## CRediT authorship contribution statement

**Kourosh Holakouie-Naieni:** Writing – review & editing, Writing – original draft, Validation, Supervision, Project administration, Formal analysis. **Mojtaba Sepandi:** Writing – review & editing, Writing – original draft, Conceptualization. **Babak Eshrati:** Methodology, Data curation. **Shahrzad Nematollahi:** Writing – review & editing, Writing – original draft, Visualization, Conceptualization. **Yousef Alimohamadi:** Writing – review & editing, Writing – original draft, Methodology, Formal analysis, Conceptualization.

## Declaration of competing interest

None of the authors have any conflict of interest to declare.
